# Conditional Seed Dormancy Helps *Silene hicesiae* Brullo & Signor. Overcome Stressful Mediterranean Summer Conditions

**DOI:** 10.3390/plants10102130

**Published:** 2021-10-07

**Authors:** Francesca Carruggio, Andrea Onofri, Stefania Catara, Carmen Impelluso, Maria Castrogiovanni, Pietro Lo Cascio, Antonia Cristaudo

**Affiliations:** 1 Department of Biological, Geological and Environmental Sciences, Germplasm Bank (BGS-CT), University of Catania, 95128 Catania, Italy; francesca.carruggio@unict.it (F.C.); scatara@unict.it (S.C.); carmen.imp@libero.it (C.I.); mariacastr.bio@gmail.com (M.C.); 2 Department of Agricultural, Food and Environmental Sciences, University of Perugia, 06121 Perugia, Italy; andrea.onofri@unipg.it; 3 Associazione Nesos, 98055 Lipari (ME), Italy; plocascio.nesos@gmail.com

**Keywords:** ex situ collection, Mediterranean germination syndrome, seed age, plant conservation, thermo-inhibition, threatened plants, seed dormancy

## Abstract

Investigations on seed biology and ecology are of major importance for the conservation of threatened plants, both providing baseline information and suggesting practical approaches. In our study, we focused on the germination behavior of *Silene hicesiae* Brullo & Signor., a narrow endemic species to Panarea and Alicudi (Aeolian Archipelago, Italy), as well as one of the 50 most threatened Mediterranean island plants. Specifically, the effects of temperature, light, seed age, seed source, and collection year were evaluated; in addition, threshold temperatures and thermal–time parameters were estimated. The thermal range for fresh seed germination resulted between 5 and 15 °C, reaching up to 20 and 25 °C at increasing seed age, with 30 °C being clearly beyond the ceiling temperature. This behavior indicates that fresh seeds exhibit the Type 1 non-deep physiological dormancy, and that germination is regulated by conditional dormancy. This dormancy syndrome emerged as a highly efficient adaptation strategy for this species and, together with thermo-inhibition, would allow seeds to counteract or take advantage of Mediterranean environmental conditions. The comparison between the wild Panarea population and the corresponding ex situ cultivated progeny has enabled the identification of the latter as a suitable seed source for sustainable in situ reinforcement actions, at least in the short-term; indeed, plant cultivation for a single generation did not produce significant modifications in the germination behavior of the offspring.

## 1. Introduction

Germination, emergence, and seedling establishment are key processes to regulate the spread and distribution of plants into changing environments [1,2,3], which is particularly important for species of conservation concern, such as rare and/or endemic species. Therefore, studying the interactions between the above processes, the genotype, and the environment appears to be particularly relevant to identify potential causes that restrict in situ germination processes [4,5] and to define efficient propagation methods. Likewise, studying the mechanisms that minimize the risk in seedling establishment and increase the probability of successful growth to maturity and reproduction [6] is fundamental, especially in arid and semiarid Mediterranean ecosystems characterized by high environmental unpredictability.

It has been shown that germination parameters (i.e., onset, capability, and speed) are strongly affected by a set of key environmental factors, such as temperature, light, and soil moisture [7,8]. Seed responses to temperature conditions differ among plant species that may show different thermal thresholds for germination. In this respect, lowland Mediterranean species typically germinate within the optimal range 5–15 °C, with germination decreasing at higher temperature values [9,10,11].

Apart from the prevailing temperature regime, daily fluctuations may also be important for germination [12,13,14], particularly in arid zones [7]. Additionally, light can have a relevant effect in inhibiting/stimulating seed germination [9,15,16]. The combined effects of fluctuating temperatures and light are particularly important in Mediterranean habitats because they may regulate the ability of seeds to emerge when buried in the soil. On the one hand, the need for dark conditions and constant temperatures may prevent the germination of seeds near to the soil surface, where moisture conditions might be limiting [9]; on the other hand, the need for light and fluctuating temperatures may be regarded as an adaptation process for small-seeded species, to ensure that germination occurs near to the soil surface in vegetation gaps [7,17].

Environmental effects are mediated by seed dormancy, which is one of the main mechanisms controlling the timing of germination [18]. Dormancy is generally considered as an adaptation to harsh environments, allowing seeds to delay germination until environmental conditions are optimal for seedling emergence, growth, and survival [19]. In some species, fresh mature seeds are not dormant, and they are able to germinate within a specific temperature range, which does not increase with time. Conversely, in other species, fresh seeds are conditionally dormant and the temperature range for germination increases with after-ripening, while non-deep physiological dormancy (non-deep PD) breaks progressively. The only way to check whether fresh seeds are non-dormant or conditionally dormant is to test them over a wide range of temperatures, soon after their harvest, and progressively, after different after-ripening periods [20]. According to seed dormancy levels, conditionally dormant seeds may vary in their sensitivity to high temperatures. Nevertheless, high thermal values may also result in so-called thermo-inhibition, by which germination is suppressed, although seeds remain viable, and germination is recovered when optimal temperature levels are re-established [5].

It is well documented that germination syndromes may greatly vary among the different populations of a species, in relation to both genetic and environmental factors. In addition, the specific conditions experienced by the mother plant during seed maturation (i.e., parental environment) may also have a great impact on the germination performance of the offspring and, in particular, on seed dormancy [14,21,22,23].

As mentioned above, investigating seed germination ecology of threatened plant species is of crucial importance, particularly for small and isolated populations; indeed, such baseline information can be used to implement efficient propagation techniques and conservation programs to counteract the risk of species extinction [24,25,26]. In this respect, maximizing the use of seeds and/or genetically variable plantlets during the implementation of in situ population reinforcement programs is one of the appropriate rules for long-term populations’ persistence [27,28]. The achievement of this goal requires sufficient amounts of viable seed available without this affecting populations’ natural regeneration. Therefore, the potential of ex situ living collections as seed sources for in situ conservation actions has been increasingly taken into account [29], even though this possibility is a hotly debated issue [30].

**Figure 1:**
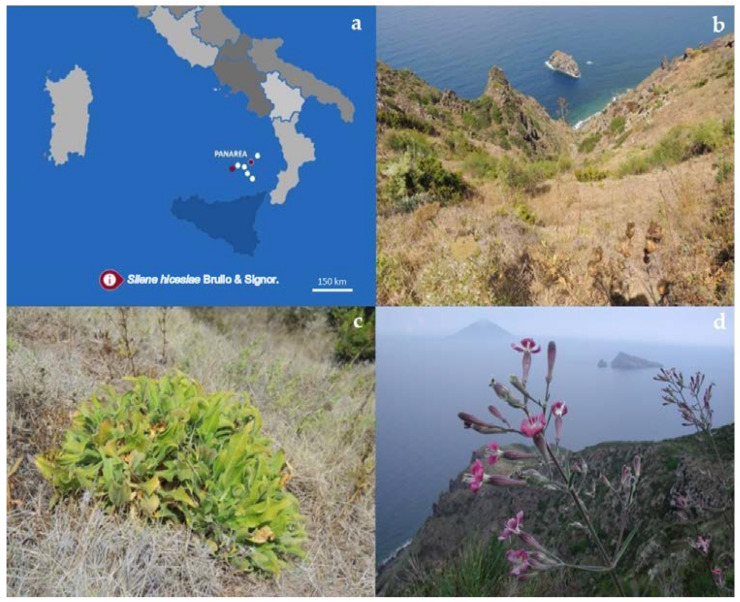
(**a**) Map of the geographic range of *S. hicesiae* (Aeolian Archipelago—Southern Italy); (**b**) growth site in Panarea; (**c**) basal rosette; (**d**) inflorescence.

In our study, we focused on *Silene hicesiae* Brullo & Signor. (Caryophyllaceae), a threatened and narrow endemic species, growing only on the steep stony slopes or cliffs of Panarea and Alicudi islands [31], two small volcanic islets of the Aeolian Archipelago (Southern Italy), about 60 km apart from each other (Figure 1).

*S. hicesiae* is included, as a priority species, in the Annexes II and IV of the European Union 92/43/EEC Habitats Directive. Additionally, it has been included by IUCN among the threatened species of the ‘Top 50 Mediterranean Island Plants’ [31].

The species is part of the ‘*Silene mollissima* group’, comprising 11 closely related species (some of which are endemic) growing mainly in coastal stands within the Western Mediterranean Basin [32,33]. According to some authors, *S. hicesiae* is closely related to *S. velutina* from Corsica [34,35]. However, a recent study has highlighted several peculiar seed traits, which the species does not share with the other congeneric taxa of the *S. mollissima* group [36]. Hence, the systematic position of *S. hicesiae* is still uncertain.

The only available information on the germination behavior of the study species is from the Seed Information Database (full germinability at 20 °C in light/dark condition, [37]). However, important information is missing, relating to the effects of environmental factors, seed source, and seed age on the main traits of seed germination syndrome, i.e., germination capability, germination speed, and dormancy. With reference to germination speed, one important trait is the germination rate (GR), which affects the ability of seeds to produce vigorous seedlings in field conditions and, thus, a better seedling establishment [38].

Therefore, we decided to fill the above knowledge gaps and laid down a set of laboratory experiments, aimed at: (i) evaluating the effect of temperature, light, seed age, and collection year on the germination behavior of *S. hicesiae*; (ii) verifying the potential presence of seed dormancy and its role in regulating the timing of germination in the wild; (iii) modelling the effect of temperature, light and seed age on GR, as well as estimating temperature thresholds and thermal–time parameters.

In order to check for the opportunity to use ex situ produced seeds for in situ conservation actions, we compared germination traits of the wild population from the Panarea Island (PAN) and its corresponding ex situ cultivated progeny (FARO). Our hypothesis was that seeds from plants under cultivation might differ in germination behavior and dormancy syndrome compared to those from wild plants, as a consequence of adaptation to artificial conditions.

## 2. Results

### 2.1. Effect of Temperature and Light on Germination Process

For the two seed sources (PAN and FARO), the final germination percentage (FGP) was significantly affected by the interaction of temperature (from 5 to 30 °C), light condition (continuous Darkness = D and alternating Light/Dark = L/D), and seed age (as days after harvest = DAH) (*p* < 0.0001).

**Figure 2:**
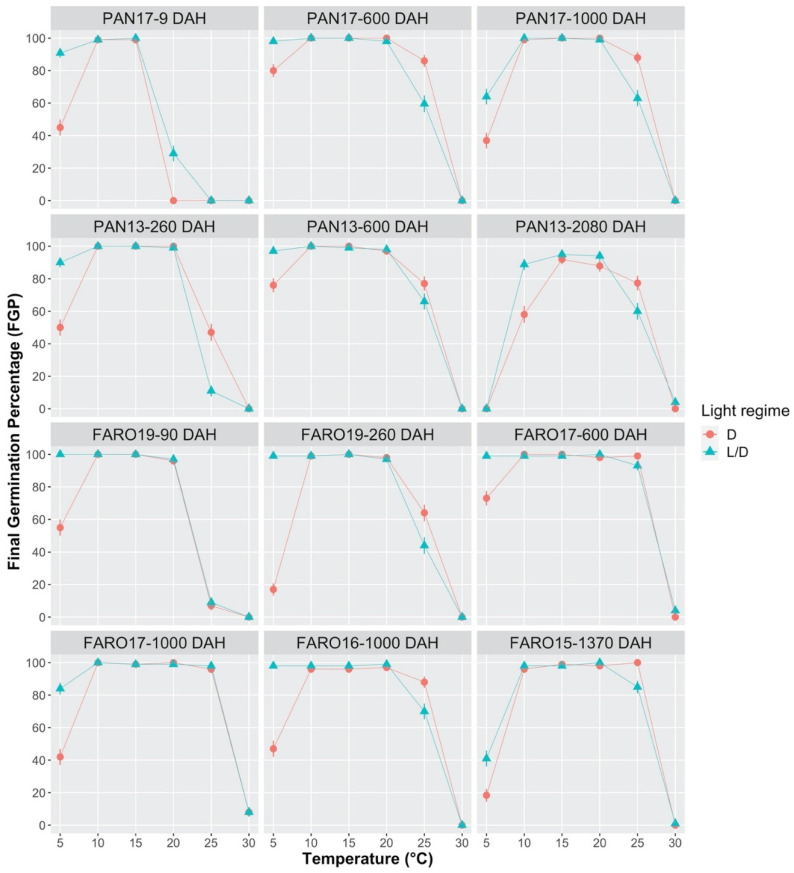
Effect of constant temperature regimes (from 5 °C to 30 °C) on the final germination percentage (FGP) of *S. hicesiae*, at seven different seed ages (9, 90, 260, 600, 1000, 1370, and 2080 days after harvest = DAH), under Light/Dark (L/D 12/12 h) and continuous Darkness (D 24 h) conditions. Seeds were collected across five years (2013, 2015, 2016, 2017, 2019) from two seed sources (PAN and FARO).

On average, temperature resulted in the highest effect and the maximum FGPs were always observed in the range from 10 to 20 °C (DAH ≥ 90). Within this range, no significant differences were found across seed sources, collection years, seed ages, and light conditions, at least within the interval 260–1000 DAH (Figure 2, Table S1). Differently, the effect of light and seed age was particularly remarkable outside this thermal range. A remarkable light-dependent effect on germination was observed at 5 °C. Indeed, seeds from both seed sources showed higher FGPs in L/D than in D (Figure 2). Moreover, a conspicuous decrease in germination performance but only at 5 °C, for both seed sources and light conditions, was detected with increasing seed age (DAH > 600; Figure 2). At the highest DAH value (i.e., PAN13–2080), germinability was null at 5 °C, whereas at 10 °C, the observed germinability was lower than at lower DAH values, indicating the possible beginning of vigor loss due to seed aging (Figure 2).

The behavior at 25 °C is particularly important, because this temperature marks the borderline between the optimal temperatures and the ceiling temperature of 30 °C, where no germination occurred. At 25 °C, for both seed sources, FGP values were low with short storage and increased remarkably as storage times increased. No significant differences within each seed source were detected between L/D and D regimes at 25 °C, apart from PAN, which showed a significantly higher performance in D than in L/D at 260 DAH (47% vs. 11%, Table S1). Moreover, the differences between collection years at 25 °C were not significant in PAN (PAN13 vs. PAN17 at 600 DAH), whereas a significant difference was observed in FARO (FARO16 vs. FARO17 at 1000 DAH in L/D regime; 70% vs. 98%). On the other hand, significant differences between seed sources under L/D conditions were observed at 25 °C, where FARO almost always performed better than PAN (Figure 2).

Fresh mature seeds (PAN17, 9 DAH), both in L/D and D conditions, were fully germinated in a narrow range of temperature values (10–15 °C). At the same time, only a small proportion of fresh seeds germinated at 20 °C (FGP = 29% in L/D; FGP = 0% in D), and no germination occurred at 25 °C. However, the thermal range for germination enlarged with increasing seed age, and germination performance improved significantly in the range 20–25 °C, regardless of the seed source (Figure 2). Specifically, seeds aged for at least 90 days provided germination percentages above 95% at 20 °C, in both seed sources and light regimes. Moreover, at least 600 days were needed to obtain FGP values over 60% at 25 °C, regardless of the seed source and light condition. Finally, we have already mentioned that no germination occurred at 30 °C in any trial. When null or low FGP values were recorded, most of the ungerminated seeds were able to recover germination if re-incubated at 15 °C (close-to-optimal temperature), under the same light conditions (recovery percentage from 76% to 100%, Table S2).

**Figure 3:**
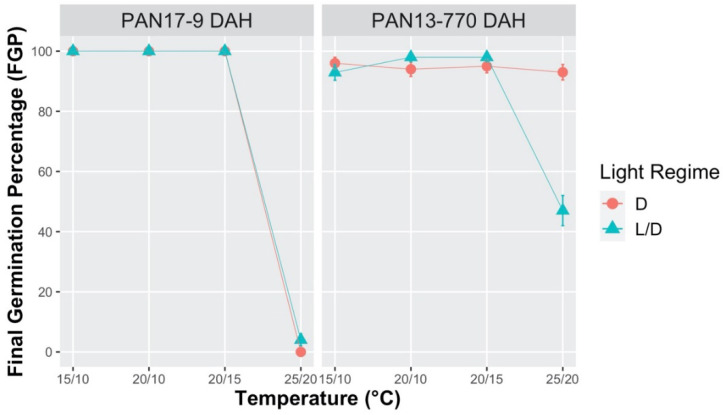
Effect of alternating temperatures (15/10 °C, 20/10 °C, 20/15 °C, and 25/20 °C), on the final germination percentage (FGP) of *S. hicesiae*, at two different seed ages (9 and 770 days after harvest = DAH), under Light/Dark (L/D 12/12 h) and continuous Dark (D 24 h) conditions. Seeds were collected across two years (2013 and 2017) from the PAN seed source.

Similar trends to those already observed at constant temperature regimes with increasing seed age were also detected under alternating temperatures in PAN (Figure 3). FGP values at 15/10, 20/10 and 20/15 °C were close to 100%, both in fresh and stored seeds (770 DAH), regardless of the photoperiod regime. In relation to fresh seeds, an inhibition effect on germination was only observed at 25/20 °C, although not at 20/15 °C, as compared to the constant regime of 20 °C, at similar seed ages. Hence, a 12 h period at 20 °C was not sufficient to inhibit germination, differently from the same length period at 25 °C.

Concerning stored seeds, no alternating regime proved to be different from the two corresponding constant regime under the same photoperiod conditions. Nevertheless, the significant difference, which was observed in FGP values between L/D and D (47% and 93%, respectively) at 25/20 °C, is notable because, as mentioned above, it was not detected in PAN for any of the constant temperature trials either at 20 °C or at 25 °C.

The germination behavior under the different thermal regimes tested indicates the presence of a dormancy status in fresh seeds, which gradually released with increasing seed age.

### 2.2. Germination Time-Course

**Figure 4:**
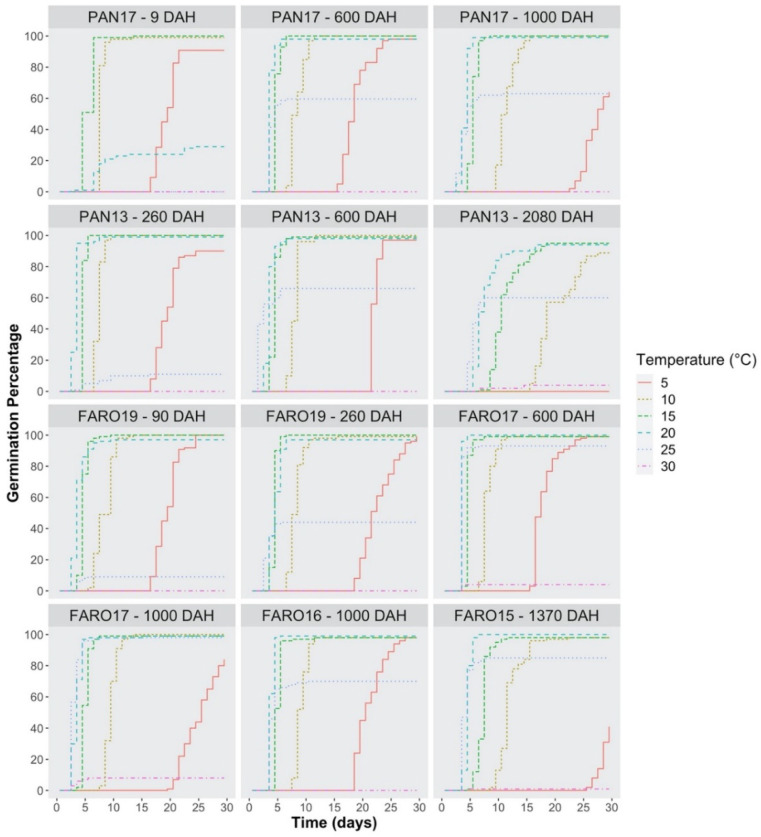
Kaplan–Meier estimates of germination curves for seeds of two *S. hicesiae* seed sources (PAN and FARO) at seven different seed ages (9, 90, 260, 600, 1000, 1370, and 2080 days after harvest = DAH), incubated for 30 days under light/dark conditions (L/D), at six constant temperatures regimes (from 5 °C to 30 °C), and expressed as the percentage of viable seeds.

The non-parametric germination functions are step curves, and they provide information on the time-course of the germination process based on the cumulative percentage of germinated seeds. Such time-courses at constant incubation temperatures highlighted a clear dependence of germination timing from both temperature and seed age (Figure 4).

The curves, for incubation temperatures in the range 15 °C to 20 °C and at seed age from 90 to 1000 DAH, showed full germination at the final inspection time, together with a very steep increase in the percentage of germinated seeds, indicating high uniformity. Otherwise, the ‘slope’ of the curves increased from 9 to 260 DAH at 20 °C, and from 260 to 600 DAH at 25 °C, which confirms that dormancy was progressively released during the storage period. In addition, a rather long lag-phase was observed at 5 °C, which became longer with increasing seed age. Likewise, the ‘slope’ at 5 °C was less steep, due to lower germination uniformity.

From the germination time-course, we can derive that 50% germination was reached in 4–5 days at 15 °C (9 ≤ DAH ≤ 1000), 3–4 days at 20 °C (90 ≤ DAH ≤ 1000) and 2–3 days at 25 °C (600 ≤ DAH ≤ 1000).

**Figure 5:**
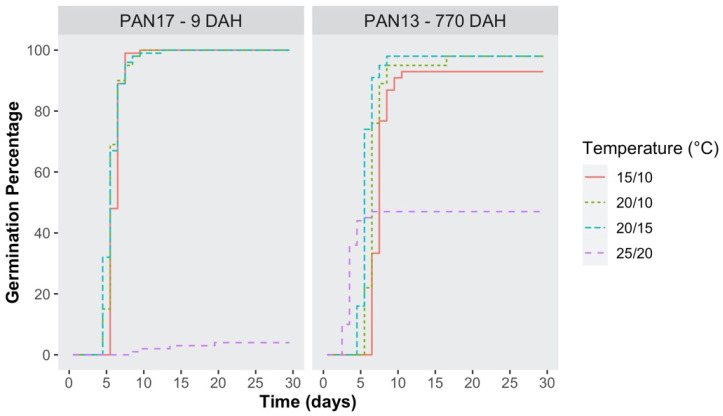
Kaplan–Meier estimates of germination curves for seeds of the *S. hicesiae* PAN seed source at two seed ages (9 and 770 days after harvest = DAH), incubated for 30 days under light/dark conditions (L/D), at four alternating temperatures (15/10 °C, 20/10 °C, 20/15 °C, and 25/20 °C), and expressed as the percentage of viable seeds.

Cumulative germination percentage curves of *S. hicesiae* under four alternating temperature regimes (15/10 °C, 20/10 °C, 20/15 °C, and 25/20 °C) are presented in Figure 5. Full germination, under the three lower alternating temperatures, was quickly reached within the ninth day in fresh seeds (PAN17, 9 DAH), whereas the germination process was slower and less uniform for seeds at 770 DAH. Conversely, at 25/20 °C, germination progress was almost negligible for fresh seeds, whereas it was quick although incomplete at 770 DAH.

### 2.3. Germination Rate, Threshold Temperatures and Thermal Requirements

**Figure 6:**
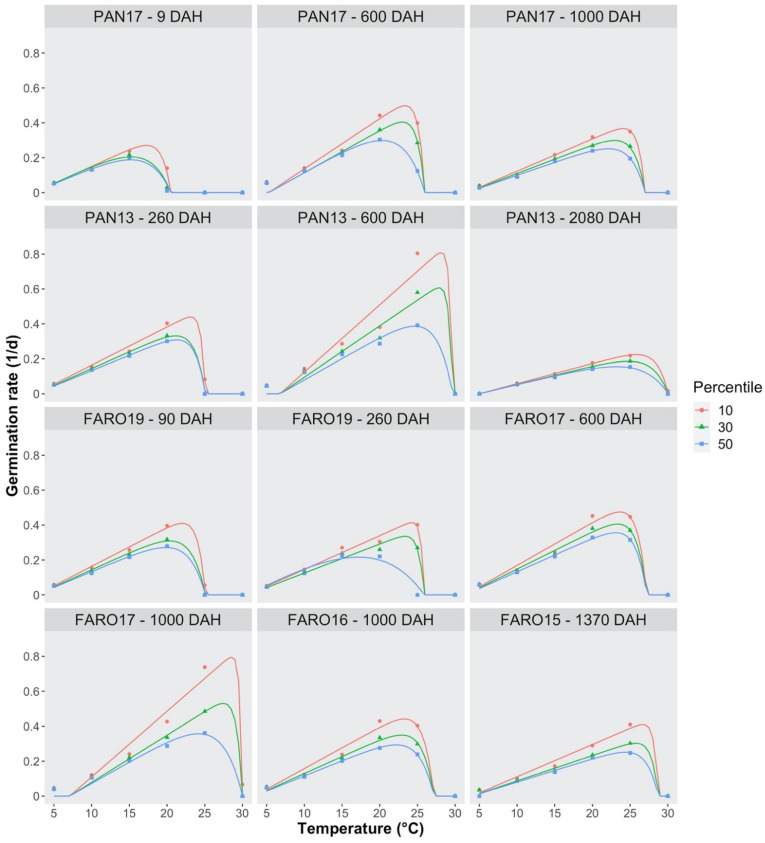
Effect of temperature on the germination rates for three germination percentiles (10th, 30th, and 50th) of *S. hicesiae* seeds, at seven seed ages (9, 90, 260, 600, 1000, 1370, and 2080 days after harvest = DAH), incubated at six constant temperatures (from 5 °C to 30 °C), under light/dark condition (L/D 12/12 h). Seeds were collected across five years (2013, 2015, 2016, 2017, and 2019) from two seed sources (PAN and FARO). The symbols show the observed data, whereas the lines show the predictions, according to Equation (1) and the parameters in Table 1.

The germination time-courses for constant temperature regimes were used to derive the times required to reach 10%, 30% and 50% germination, whose reciprocal values are the germination rates for the 10th, 30th and 50th percentiles (GR_10_, GR_30_ and GR_50_). For each percentile, the GR values were regressed against temperature, by using an exponential model (see Equation (1) in *Data Analysis*). The model shows that the GRs are 0 at the base temperature level (T_b_), which is common to all percentiles within each seed lot. At sub-optimal temperature levels, GR values increased steadily, reaching a maximum value at the optimal temperature for germination (T_o_) (Figure 6), which was different, depending on the percentile. At super-optimal temperatures, GR values decreased, reaching 0 at the ceiling temperature (T_c_), which was common to all percentiles.

The maximum GR value at the optimal temperature increased with seed age. Considering the 10th percentile, the value was lower than 0.3 d^−1^ at 9 DAH (PAN17) and increased up to 0.5 d^−1^ in PAN17 and to 0.8 d^−1^ in PAN13 at 600 DAH. At higher seed ages (1000 and 2080 DAH), a noticeable decrease in GR was detected, down to less than 0.4 d^−1^ in PAN17 and to approximately 0.2 d^−1^ in PAN13.

The trend for FARO was less clear; indeed, the GR values were around 0.4 regardless of DAHs, except for FARO17 at 1000 DAH (Figure 6).

Thermal times to reach 10%, 30% and 50% germination (θ_T_, °Cd) and the estimated threshold temperatures are presented in Table 1. In particular, it was noted that θ_T_ changed with seed age, increasing from 54.0 °Cd to 67.0 °Cd in PAN17, when DAH increased from 9 to 1000, and from 56.7 °Cd to 96.5 °Cd in PAN13, when DAH rose from 260 to 2080. A similar change was also observed in FARO (Table 1).

As DAH increased, the T_c_ as well as the T_o_ was raised for both seed sources. The T_c_ was about 20.5 °C in seeds at 9 DAH and reached 25.1 °C at 260 DAH. Accordingly, the T_o_ for the 50th percentile varied from 21.3 °C at 260 DAH, to 24.6 °C at 600 DAH, and to 23.2 °C at 2080 DAH (in PAN13), and from 15.2 °C at 9 DAH, to 20.3 °C at 600 DAH, and to 22.1 °C at 1000 DAH (in PAN17) (Table 1). Moreover, T_o_ varied among germination percentiles: the lowest percentile (10th) had higher T_o_ values, compared with the highest percentile (50th), which showed lower T_o_ values (Table 1). Conversely, the T_b_ did not change within 260 DAH, although increased for higher DAH values (6.8 °C and 5.3 °C at 600 DAH, in PAN13 and PAN17, respectively).

**Figure 7:**
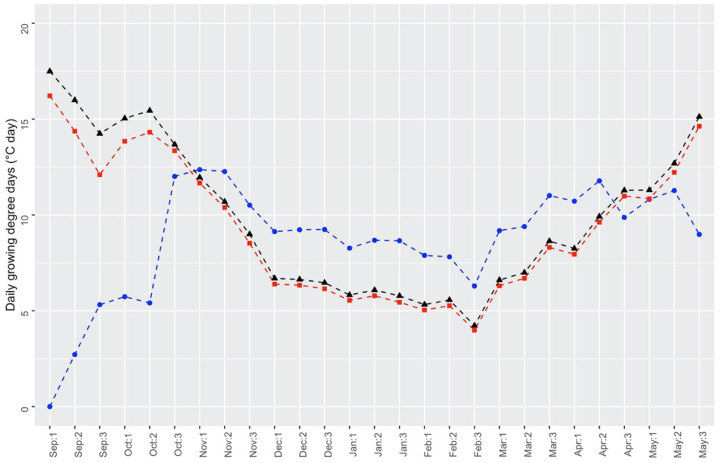
Thermal time accumulated in one day, at the beginning of each decade from September to May. The blue line with circles refers to 9 DAH seeds (DAH = days after harvest), the red line with squares refers to 600 DAH seeds, and the black line with triangles refers to 2080 DAH seeds.

The thermal time model was also used to estimate the daily accumulation of thermal time in the wild Equation (2), based on the daily average air temperatures recorded from 2012 to 2018 at the climatic station of Lipari, which is the nearest to the Panarea island. Figure 7 shows that the daily accumulation of thermal time for seeds dispersed in July can give them a good chance to start the germination process between the third decade of October and the first decade of November, whereas earlier emergences are prevented from high temperature levels (>T_c_, from September to the second decade of October). Once the germination has started, it can progress quickly, reaching a value of 50% in about 4.5 days (12.5 °Cd for an estimated thermal time of 55 °Cd, at 90 DAH; see Table 1). The inhibition of germination during late summer/early autumn for seeds at 600 and 2080 DAH is much less marked (Figure 7).

## 3. Discussion

In the present study, the germination behavior of *S. hicesiae* was investigated, evaluating the effects of temperature, light, seed age, seed source, collection year, and their interactions. The relationship between germination rate and temperature was considered, to estimate cardinal temperatures as well as thermal requirements for germination. The possible ‘cultivation effect’ (i.e., adaptation to artificial conditions) on germination patterns was also explored by comparing both a wild population (PAN) and its cultivated progeny (FARO), checking this last one as a suitable seed source for conservation purposes.

Fresh mature seeds of *S. hicesiae* (9 DAH) only germinated over a narrow range of temperatures (5–15 °C), both in L/D and D conditions, whereas they did not germinate at temperatures equal to or higher than 25 °C. In addition, the thermal range for germination enlarged with seed age, reaching up to 20 °C and 25 °C which is the possible maximum for this genotype, whereas 30 °C was clearly beyond the ceiling temperature. This behavior indicates that fresh seeds of *S. hicesiae* exhibit the Type 1 non-deep PD [20,39] and that germination is regulated by conditional dormancy (CD). Furthermore, fresh seeds proved full germination capacity (100%) at 10 °C and 15 °C, regardless of light conditions, and at 5 °C under a L/D regime; consequently, it can be concluded that the seeds of *S. hicesiae* are already conditionally dormant when they are dispersed by mother plants, and they do not pass through a full dormancy state. Such a CD model pattern has been reported, for instance, in *Capsella bursa-pastoris* [20,40]. Accordingly, only the range of cardinal temperatures changed, with T_c_ and T_o_ both increasing and T_b_ remaining constant (around 2.5 °C), while CD was releasing. This behavior arises from the progression of a dormancy continuum [20], i.e., the gradual physiological sequence of transitional states, which conditionally dormant seeds of *S. hicesiae* undergo during non-deep PD release. At increasing DAHs, seeds become physiologically able to germinate under higher temperatures, thus exhibiting higher T_c_ values.

*S. hicesiae* seeds are dispersed during summer and they overcome the typical hot Mediterranean conditions through a double strategy. More explicitly, with fresh seeds, thermo-inhibition prevents germination at hot temperatures (T_c_ is below 30 °C; see Table 1), whereas at lower temperature values (20–25 °C), the lack of germination results from CD. Together, these mechanisms provide efficient protection from ill-timed germination, following both sporadic summer rain events (thermo-inhibition) and early autumn rain events (conditional dormancy). Therefore, germination is delayed until a reliable soil moisture availability is ensured in late autumn. This is in accordance with the fact that thermo-dormancy was not induced, as demonstrated by seeds remaining viable when exposed to unfavorable high temperatures and being able to recover full germination ability, when exposed to optimal thermal values (Table S2), which has previously been found in other Mediterranean species [5].

Germination at low temperatures (10–15 °C or 10–20 °C) is a common trait in lowland Mediterranean plant species, according to a convergent evolutionary process defined as ‘Mediterranean germination syndrome’ [5,10,41,42,43]. For instance, Zani and Müller [44], found a similar thermal preference in other species of the *Silene* genus from Mediterranean climates, as did Murru et al. [32,33] in other Mediterranean species of the *S. mollissima* group, three of which are clearly lowland plants. Our trials also suggest that *S. hicesiae* seeds can germinate both on the soil surface and when slightly buried, under optimal temperatures. This result does not confirm the data obtained at 15 °C with three costal species of the *S. mollissima* group (i.e., *S. velutina*, *S. badaroi*, and *S. hichnusae*) that showed a preference for light [32].

Under optimal temperature conditions, *S. hicesiae* seeds are also able to fully germinate within a few days, and quite synchronously (Figure 4). Such a relatively rapid germination (approximately 4 days at 15 °C and 20 °C) might be an advantageous trait for successful seedling establishment, over more slowly germinating species. Germination speed considerably decreased only at low temperature values (5 °C), under which seeds also showed improved germination in the presence of light. This should imply better establishment opportunities when the seeds are close to the soil surface, where early growing stages may benefit from solar radiation, with a very low risk of frost during Mediterranean winters.

A low germination speed is considered as a typical trait of Mediterranean species, preventing seed cohort loss due to autumn uneven rainfalls [42,45]. However, other perennial coastal species from the Mediterranean area have even faster germinating seeds compared to *S. hicesiae* [46,47]. Interestingly, both slow and rapid germinators have been detected across Mediterranean species of the *Silene* genus [44]. Therefore, germination speed should possibly be reconsidered within the wider context of different germination/dormancy syndromes observed across Mediterranean species. For instance, based on our results, a relatively rapid germination might also be a sustainable trait for lowland Mediterranean species, if they may widely count on Type 1 non-deep PD associated with CD.

As far as we know, there is no availability of systematic literature data about the presence of CD in Mediterranean species, although see [32,48,49,50]. Nevertheless, several authors have generically reported that PD in seeds of herbaceous Mediterranean species is broken during dry storage at 20 °C or higher [18]. Therefore, future specific surveys should elucidate whether the Type 1 non-deep PD should also be considered as a widespread trait in Mediterranean species.

Our thermal time model estimated germination onset in *S. hicesiae* in the wild by the third decade of October. Based on the species’ seed behavior (CD, thermo-inhibition effect, optimal thermal germination range, lack of light preference and fast germination in the same range, as well as relatively fast dormancy loss), it could be supposed that almost the entire annual seed production could possibly germinate in the autumn–winter following seed dispersal. Although some germinations might also occur in the following spring, our results suggest that *S. hicesiae* could, at most, develop a transient soil seedbank *sensu* Walck et al. [51], because all seeds are expected to ‘disappear’ from the soil before the second germination season. The lack of a persistent soil seedbank may hinder the ability of *S. hicesiae* to overcome unpredictable and extreme environmental events [52,53,54]. However, the perennial life cycle might help maintain wild populations of *S. hicesiae*, because each individual can act as a multiple-year seed source.

Regarding the potential ‘cultivation effect’ on seed germination behavior, our findings showed that seeds from both sources (PAN and FARO) did not differ either in their germination behavior or in their dormancy syndrome pattern. Specifically, seeds from cultivated plants retained CD and exhibited thermo-inhibition as well, with both events being of major importance within the perspective of using FARO seeds for population reinforcement projects in the wild. Some differences between the two seed sources concerned only FGP values at 25 °C in L/D conditions, with FARO showing higher values (i.e., lower dormancy levels). Lower seed dormancy and higher germination rates are well documented traits, not only in cultivated agricultural species and garden varieties [55,56,57,58,59], but also in wild plants cultivated in botanic gardens, or in commercial nurseries within habitat restoration projects [60,61]. Therefore, it is interesting that only minor differences emerged in this study case. Their small extents are probably related to both the perennial life cycle of *S. hicesiae* and the single plant generation from which all of the FARO seed lots were collected. Indeed, annual and biennial species have been found more prone to cultivation effects as compared to perennials [62], and the increase in the number of generations of ex situ living collections is considered as a possible cause of undesired shifts in biological traits under artificial selection [30].

## 4. Materials and Methods

### 4.1. Seed Lot Details

Mature capsules of *S. hicesiae* were collected at the time of natural seed dispersal from the Panarea wild population (PAN) (338 m a.s.l., N 38°38′26.14′′, E 15°03′49.08′′, Panarea Island, Lipari, Messina), by sampling 50 randomly selected mother plants. Seed collection was carried out with the permission of the Sicilian Regional Authority for Territory & Environment. Other mature fruits were collected from 30 individuals of an ex situ living plant collection (FARO), established in 2014 at the ‘Piante Faro’ private nursery (103 m a.s.l., N 37°41′47.55′′, E 15°11′25.23′′, Giarre, Catania). To set up this ex situ living plant collection, seeds were originally collected in 2013 from the Panarea population. The plants were cultivated in pots (18 cm diameter) under open-field conditions. Water irrigation was supplied from May to August for 10 min every two days, using a water sprinkler system.

After collection, fruits were cleaned, and seeds were stored in paper bags at room temperature (22 °C ± 2 °C) until germination experiments started.

### 4.2. Seed Germination Behavior Assessment

Germination behavior of fresh and dry stored seeds was evaluated under laboratory conditions at the Germplasm Bank (BGS-CT) of the University of Catania (Italy). Seed lots from PAN and FARO were compared from different collection years and at different seed ages (Table 2).

To assess the sensitivity to temperature, light, and seed age of *S. hicesiae* seeds, germination assays were performed at six constant (5 °C, 10 °C, 15 °C, 20 °C, 25 °C, and 30 °C) and four alternating (15/10 °C, 20/10 °C, 20/15 °C, and 25/20 °C; thermoperiod 12/12 h) temperature regimes (Table 2). Constant temperatures values were chosen to include both optimal germination temperatures for Mediterranean plants (10–20 °C) while including cold and hot temperatures: 5 °C and 30 °C, respectively. Fluctuating temperature regimes were selected considering the values that are normally experienced in natural conditions during the supposed germination season (autumn–early spring) near the soil surface. Each thermal condition was tested both in continuous Darkness (D) and in alternating Light/Dark conditions (L/D) with a 12/12 h photoperiod, simulating seeds being below and above the soil surface. In the alternating temperature experiments, the exposure to light coincided with the highest temperature to replicate day/night temperatures.

Germination assays were performed in 9 cm Petri dishes containing three layers of filter paper moistened with 5 ml of distilled water. For each treatment, four Petri dishes, each with 25 seeds, were used. The Petri dishes were placed in germination chambers (Sanyo—model MLR-351H, Tokyo, Japan), equipped with cool white fluorescent tubes (Osram FL 40 SS W/37, München, Germany). For treatments in darkness, Petri dishes were immediately wrapped in two layers of aluminum foil. All Petri dishes were sealed with Parafilm M® to prevent moisture loss and water was added to the dishes as needed to maintain an adequate moisture level. To ensure no systematic effect due to the positioning within the growth chamber, Petri dishes were re-randomized every 2 days. Seeds incubated in the light were counted daily and germinated seeds were discarded, whereas dark-incubated seeds were counted only once at the end of the test to avoid any exposure to light.

A seed was considered germinated when the radicle was at least 1 mm long. Germination tests lasted 30 days, after which ungerminated seeds were checked for viability using a cut test. Seeds with a firm and white embryo were considered viable. The final germination percentage (FGP± SE) was calculated on the basis of the total number of filled seeds.

To investigate whether low FGP values, which were recorded in some cases at 5 °C, 20 °C, 25 °C, and 30 °C incubation temperature, were due to seed thermo-inhibition, thermo-dormancy or viability loss, recovery experiments were performed after 10 days without additional germination. For this purpose, ungerminated seeds were transferred to 15 °C, for two additional weeks (recovery period). After this period, further germination (%) was calculated. All remaining ungerminated seeds were tested for their viability by cut tests.

### 4.3. Data Analysis

The observed final germination percentage (FGP) was modelled by fitting generalized linear models (GLMs) and including the experimental variables (temperature, light, seed age, seed source, and collection year) as the explanatory factors.

In order to assess germination speed, the counts of germinated seeds were used to estimate non-parametric germination curves, by utilizing the Kaplan–Meier (KM) method, as implemented in the ‘Survfit’ function in R [63]. The KM method belongs to the body of survival analyses, and it has been successfully used to model seed germination [64,65] because it enables the expression of the proportion of seeds germinated/seedlings emerged as a function of time without reference to any predetermined equation. KM curves were used to derive the time for 10%, 30% and 50% germination, whose reciprocals were taken as the germination rates (GRs, in 1/d) for the corresponding percentile (GR_10_, GR_30_ and GR_50_). Following Bradford [66], percentiles were defined considering the whole seed population and not only the germinated fraction; for the cases where a certain germination percentage was not attained, the corresponding GR was considered to be 0.

Considering the two populations and the different DAHs separately, GRs were used to parameterize the following thermal–time model:

(1) $$\mathrm{GRg}=\frac{{T-T}_{b}}{\theta_{\left(g,T\right)}}\left\{\frac{1-\exp\left[k_{\left(g\right)}\left({T-T}_{c}\right)\right]}{1-\exp\left[k_{\left(g\right)}\left(T_{b}{-T}_{c}\right)\right]}\right\}$$

where GRg is the germination rate for the gth percentile (i.e., GR_10_, GR_30_ and GR_50_), expressed as a function of the environmental temperature T, T_b_ is the base temperature, T_c_ is the ceiling temperature, θ_(g,T)_ is the thermal time to germination, depending on the temperature and germination percentile, and k_(g)_ is a regression parameter, describing the shape of the relationship between germination rate and temperature, depending on the percentile g. The above equation works under the constraint that GRg is 0 for T < T_b_ and T > T_c_. Preliminary analyses based on the Akaike information criterion [67] showed that the threshold temperatures T_b_ and T_c_ were constant and independent from the percentiles. Optimal temperatures (T_o_) were derived from the above equation, by equating the first derivative to 0 and finding the root within the interval from T_b_ and T_c_.

In the below equation, the daily accumulation of thermal time (TT), depending on temperature (at sub-optimal and super-optimal values) and percentile, is obtained as:

(2) $$\mathrm{TT}=\left({T-T}_{b}\right)\left\{\frac{1-\exp\left[k_{\left(g\right)}\left({T-T}_{c}\right)\right]}{1-\exp\left[k_{\left(g\right)}\left(T_{b}{-T}_{c}\right)\right]}\right\}$$

We used Equation (2) to provide at least a general estimate of the time to 50% germination in the wild, based on the daily average air temperatures recorded from 2012 to 2018 at the climatic station of Lipari [68], which is the nearest to the experimental site (19.31 km apart from Panarea). Daily TTs were calculated from the beginning of September, because germinations are usually impaired before that time, due to water shortages.

All analyses were performed in the R statistical environment [69].

## 5. Conclusions

Our findings enabled the definition of the general frame for germination behavior in *S. hicesiae*. The Type 1 non-deep PD, detected in this study, seems to fit the climatic conditions of the lowland Mediterranean areas, supporting the hypothesis that this dormancy syndrome may be much more widespread than actually noticed among the species growing in those areas. Future research should be planned to elucidate this.

Some interesting elements also emerged from a conservation perspective, on both the ex situ and the in situ conservation side. For instance, seeds stored at room conditions might be used for several years (at least for five years), thanks to their long-lasting viability and germination capacity, both to obtain new individuals to be transplanted in the wild and to establish ex situ living collections, lasting at least one generation, from which seeds could be obtained for in situ population reinforcements. Such interventions should be planned, especially on the Alicudi Island, where a few tens of spontaneous individuals grow, but also on Panarea where, according to our recent surveys, the existing population amounts to <500 individuals. Both the direct sowing of dry stored seeds and transplanting in autumn or early winter would be suitable intervention techniques. Ex situ germplasm management, in seed banks and nurseries, would result in a reduced collection pressure on wild populations during conservation plans.

Finally, other research issues are still open. Re-sowing trials for seeds collected from cultivated plants are needed to assess the germination behavior of seeds from subsequent generations, to enhance the life span of ex situ living collections. Apart from germination traits, other traits should also be evaluated for later generations, such as decreases in stress tolerance, which might be modified under artificial selection pressure [70]. Other relevant traits deserving further research attention in the wild might be the average generation time, soil seed bank ecology, and inter-specific relationships (e.g., seed predation and plant-to-plant competition in early developing stages). For instance, relevant information could be provided by in situ burial experiments combined with soil temperature and humidity data recording.

## Figures and Tables

**Table 1:** Threshold temperatures and thermal requirements for *S. hicesiae* PAN and FARO seed sources, with standard errors in parentheses, at different seed ages (days after harvest = DAH). No differences across percentiles were found for base temperature and ceiling temperature. T_b_, base temperature; T_c_, ceiling temperature; T_o_, optimal temperature; θ_T_, thermal time to germination for the corresponding percentile.

Seed Lot ID	Seed Age DAH	Parameters							
		T_b_ (°C)	T_c_ (°C)	T_o_ (°C)			θ_T_ (°Cd)		
				10th	30th	50th	10th	30th	50th
PAN17	9	2.35 (0.79)	20.52 (0.17)	17.3 (0.91)	15.2 (1.44)	15.2 (1.56)	50.92 (5.24)	50.32 (9.67)	54.02 (12.2)
PAN17	600	5.27 (0.67)	25.98 (0.38)	23.4 (1.53)	23.0 (1.82)	20.3 (1.33)	34.74 (2.33)	41.46 (3.12)	41.23 (5.62)
PAN17	1000	3.36 (0.70)	26.99 (3.11)	24.1 (9.30)	23.0 (7.97)	22.1 (7.77)	54.10 (4.73)	60.76 (5.03)	67.03 (10.5)
PAN13	260	2.50 (0.58)	25.08 (0.04)	23.2 (2.57)	21.2 (1.97)	21.3 (4.93)	45.77 (2.61)	52.04 (3.99)	56.66 (5.31)
PAN13	600	6.77 (0.36)	29.67 (0.18)	28.2 (9.97)	27.9 (8.58)	24.6 (2.53)	25.99 (0.86)	34.02 (1.23)	40.87 (3.84)
PAN13	2080	4.97 (0.85)	30.06 (0.09)	25.9 (7.66)	24.7 (2.23)	23.2 (1.61)	86.66 (12.3)	94.92 (12.7)	96.52 (16.0)
FARO19	90	2.66 (0.45)	25.09 (0.03)	21.8 (0.91)	20.3 (0.77)	19.9 (0.70)	44.89 (2.08)	51.03 (3.49)	55.07 (4.32)
FARO19	260	2.48 (0.49)	25.90 (0.21)	24.3 (0.45)	23.4 (0.64)	17.2 (0.88)	51.64 (2.36)	60.10 (3.11)	45.40 (5.84)
FARO17	600	3.13 (0.37)	27.20 (0.80)	23.7 (1.50)	23.4 (1.59)	23.1 (1.76)	40.71 (1.63)	46.56 (2.04)	51.94 (2.50)
FARO17	1000	7.08 (0.30)	30.02 (0.21)	28.6 (0.34)	27.5 (1.98)	24.2 (0.67)	26.61 (0.75)	37.03 (1.18)	40.49 (2.68)
FARO16	1000	3.36 (0.40)	27.19 (0.74)	23.3 (1.26)	22.9 (1.68)	22.2 (1.42)	42.09 (1.82)	51.38 (2.69)	57.43 (3.55)
FARO15	1370	4.02 (0.46)	28.74 (7.85)	26.7 (11.1)	25.8 (11.5)	24.4 (10.8)	54.05 (2.16)	68.76 (3.68)	74.80 (5.72)

**Table 2:** Experimental design of the germination tests conducted in the laboratory: DAH = days after harvest.

Seed Source	Seed Lot ID	Collection Date	Seed Age DAH	Incubation Temperature (°C)	Light Conditions
Panarea (PAN)	PAN13	July 2013	260	5, 10, 15, 20, 25, 30	Light/Dark (12/12 h) Darkness (24 h)
			600		
			770	15/10, 20/10, 20/15, 25/20	
			2080	5, 10, 15, 20, 25, 30	
	PAN17	July 2017	9	5, 10, 15, 20, 25, 30 15/10, 20/10, 20/15, 25/20	
			600	5, 10, 15, 20, 25, 30	
			1000		
Faro nursery (FARO)	FARO15	July 2015	1370	5, 10, 15, 20, 25, 30	Light/Dark (12/12 h) Darkness (24 h)
	FARO16	July 2016	1000		
	FARO17	July 2017	600		
			1000		
	FARO19	July 2019	90		
			260		

## Data Availability

Data are available from the authors upon request.

## Supplementary Materials

The following is available online at https://www.mdpi.com/article/10.3390/plants10102130/s1, Table S1: Final germination percentage (FGP) with standard error (SE) for all experimental treatments. Means with no significance letters in common are significantly different (*p* < 0.05). DAH, days after harvest, Table S2: Recovery trials. The seeds ungerminated under the first incubation temperature (T1 = 5 °C, 20 °C, 25 °C and 30 °C) were re-incubated, under the same light conditions, at the optimal temperature (T2 = 15 °C). DAH, days after harvest; FGP, final germination percentage at T1, T2 and their sum (T1 and T2); SE, standard error.

## References

[1] Ganatsas P., Tsakaldimi M., Damianidis C., Stefanaki A., Kalapothareas T., Karydopoulos T., Papapavlou K. (2019). Regeneration ecology of the rare plant species *Verbascum dingleri*: Implications for species conservation. Sustainability.

[2] Zanetti M., Dayrell R.L.C., Wardil M.V., Damasceno A., Fernandes T., Castilho A., Santos F.M.G., Silveira F.A.O. (2020). Seed functional traits provide support for ecological restoration and ex situ conservation in the threatened Amazon ironstone outcrop flora. Front. Plant Sci..

[3] Walter G.M., Catara S., Bridle J.R., Cristaudo A. (2020). Population variation in early development can determine ecological resilience in response to environmental change. New Phytol..

[4] Schemske D.W., Husband B.C., Ruckelshaus M.H., Goodwillie C., Parker I.M., Bishop J.G. (1994). Evaluating approaches to the conservation of rare and endangered plants. Ecology.

[5] Cristaudo A., Catara S., Mingo A., Restuccia A., Onofri A. (2019). Temperature and storage time strongly affect the germination success of perennial *Euphorbia* species in Mediterranean regions. Ecol. Evol..

[6] Harper J.L. (1977). Population Biology of Plants.

[7] Probert R.J., Fenner M. (2000). The role of temperature in the regulation of seed dormancy and germination. Seeds: The Ecology of Regeneration in Plant Communities.

[8] Gresta F., Cristaudo A., Onofri A., Restuccia A., Avola G. (2010). Germination response of four pasture species to temperature, light, and post-harvest period. Plant Biosyst..

[9] Thanos C.A., Georghiou K., Skarou F. (1989). *Glaucium flavum* seed germination: An ecophysiological approach. Ann. Bot..

[10] Thanos C.A., Kadis C.C., Skarou F. (1995). Ecophysiology of germination in the aromatic plants thyme, savory and oregano (Labiatae). Seed Sci. Res..

[11] Tlig T., Gorai M., Neffati M. (2008). Germination responses of *Diplotaxis harra* to temperature and salinity. Flora.

[12] Bradford K.J. (2005). Threshold models applied to seed germination ecology. New Phytol..

[13] Wang W.Q., Cheng H.Y., Song S.Q. (2013). Development of a threshold model to predict germination of *Populus tomentosa* seeds after harvest and storage under ambient condition. PLoS ONE.

[14] Cristaudo A., Gresta F., Catara S., Mingo A. (2014). Assessment of daily heat pulse regimes on the germination of six *Amaranthus* species. Weed Res..

[15] Thanos C.A., Georghiou K., Douma D.J., Marangaki C.J. (1991). Photoinhibition of seed germination in Mediterranean maritime plants. Ann. Bot..

[16] Thanos C.A., Georghiou K., Delipetrou P. (1994). Photoinhibition of seed germination in the maritime plant *Matthiola tricuspidata*. Ann. Bot..

[17] Catara S., Cristaudo A., Gualtieri A., Galesi R., Impelluso C., Onofri A. (2016). Threshold temperatures for seed germination in nine species of *Verbascum* (Scrophulariaceae). Seed Sci. Res..

[18] Baskin C.C., Baskin J.M. (2014). Seeds: Ecology, Biogeography, and Evolution of Dormancy and Germination.

[19] Cristaudo A., Gresta F., Restuccia A., Catara S., Onofri A. (2016). Germinative response of redroot pigweed (*Amaranthus retroflexus* L.) to environmental conditions: Is there a seasonal pattern?. Plant Biosyst..

[20] Soltani E., Baskin C.C., Baskin J.M. (2017). A graphical method for identifying the six types of non-deep physiological dormancy in seeds. Plant Biol..

[21] Andersson L., Milberg P. (1998). Variation in seed dormancy among mother plants, populations and years of seed collection. Seed Sci. Res..

[22] Gutterman Y., Fenner M. (2000). Maternal effects on seeds during development. Seeds: The Ecology of Regeneration in Plant Communities.

[23] Donohue K. (2009). Completing the cycle: Maternal effects as the missing link in plant life histories. Philos. Trans. R. Soc. B Biol. Sci..

[24] Godefroid S., Le Pajolec S., Van Rossum F. (2016). Pre-translocation considerations in rare plant reintroductions: Implications for designing protocols. Plant Ecol..

[25] Iralu V., Barbhuyan H.S.A., Upadhaya K. (2019). Ecology of seed germination in threatened trees: A review. Energy Ecol. Environ..

[26] Saatkamp A., Cochrane A., Commander L., Guja L., Jimenez-Alfaro B., Larson J., Nicotra A., Poschlod P., Silveira F.A.O., Cross A. (2019). A research agenda for seed-trait functional ecology. New Phytol..

[27] Basey A.C., Fant J.B., Kramer A.T. (2015). Producing native plant materials for restoration: 10 rules to collect and maintain genetic diversity. Nativ. Plants J..

[28] Volis S. (2019). Conservation-oriented restoration—A two for one method to restore both threatened species and their habitats. Plant Divers..

[29] Volis S. (2017). Complementarities of two intermediate conservation approaches. Plant Divers..

[30] Ensslin A., Godefroid S. (2019). How the cultivation of wild plants in botanic gardens can change their genetic and phenotypic status and what this means for their conservation value. Sibbaldia.

[31] Pasta S., Perez-Graber A., Fazan L., de Montmollin B. (2017). The Top 50 Mediterranean Island Plants UPDATE 2017.

[32] Murru V., Santo A., Piazza C., Hugot L., Bacchetta G. (2015). Seed germination, salt-stress tolerance, and the effect of nitrate on three Tyrrhenian coastal species of the Silene mollissima aggregate (Caryophyllaceae). Botany.

[33] Murru V., Santo A., Gallo M., Cardona C., Boi M., Bacchetta G. (2017). Comparative germination ecology and seedling growth of two Ibero-Levantine endemic species belonging to the *Silene mollissima* aggregate (Caryophyllaceae). Flora.

[34] Brullo S., Signorello P. (1984). *Silene hicesiae*, a new species from Aeolian Islands. Willdenowia.

[35] Jeanmonod D. (1984). Révision de la section Siphonomorpha Otth du genre *Silene* L. (Caryophyllaceae) en Méditerranée occidentale II: Le groupe de *S. mollissima*. Candollea.

[36] Murru V., Grillo O., Santo A., Ucchesu M., Piazza C., Gaio A., Carta A., Bacchetta G. (2019). Seed morpho-colorimetric analysis on some Tyrrhenian species of the *Silene mollissima* aggregate (Caryophyllaceae). Flora.

[37] Royal Botanic Gardens Kew. Seed Information Database (SID). Version 7.1 (May 2008). https://data.kew.org/sid/SidServlet?ID=46635&Num=813#Germination.

[38] Aldhous J.R. (1972). Nursery Practice.

[39] Schütz W., Milberg P., Lamont B.B. (2002). Seed dormancy, after-ripening and light requirements of four annual Asteraceae in south-western Australia. Ann. Bot..

[40] Baskin J.M., Baskin C.C. (1989). Germination responses of buried seeds of Capsella bursa-pastoris exposed to seasonal temperature changes. Weed Res..

[41] Thompson P.A. (1970). Germination of species of Caryophyllaceae in relation to their geographical distribution in Europe. Ann. Bot..

[42] Doussi M.A., Thanos C.A. (2002). Ecophysiology of seed germination in Mediterranean geophytes. 1. *Muscari* spp.. Seed Sci. Res..

[43] Perez-Garcia F., Hornero J., Gonzalez-Benito M.E. (2003). Interpopulation variation in seed germination of five Mediterranean Labiatae shrubby species. Isr. J. Plant Sci..

[44] Zani D., Müller J.V. (2017). Climatic control of germination in the genus *Silene* L.. Plant Ecol. Divers..

[45] Picciau R., Pritchard H., Mattana E., Bacchetta G. (2019). Thermal thresholds for seed germination in Mediterranean species are higher in mountain compared with lowland areas. Seed Sci. Res..

[46] Cogoni D., Mattana E., Fenu G., Bacchetta G. (2012). From seed to seedling: A critical transitional stage for the Mediterranean psammophilous species *Dianthus morisianus* (Caryophyllaceae). Plant Biosyst..

[47] De Vitis M., Seal C.E., Ulian T., Pritchard H.W., Magrini S., Fabrini G., Mattana E. (2014). Rapid adaptation of seed germination requirements of the threatened Mediterranean species *Malcolmia littorea* (Brassicaceae) and implications for its reintroduction. S. Afr. J. Bot..

[48] Copete M.Á., Herranz J.M., Ferrandis P. (2009). Seed germination ecology of the endemic Iberian winter annuals *Iberis pectinata* and *Ziziphora aragonensis*. Seed Sci. Res..

[49] Carta A., Hanson S., Müller J.V. (2016). Plant regeneration from seeds responds to phylogenetic relatedness and local adaptation in Mediterranean *Romulea* (Iridaceae) species. Ecol. Evol..

[50] Visser M., Beaugendre A. (2019). Conditional dormancy of *Stipa lagascae* (Poaceae) bulk-harvested on seed increase plots in South Tunisia: A reassessment and a surprise. Plant Ecol. Evol..

[51] Walck J., Baskin J., Baskin C., Hidayati S. (2005). Defining transient and persistent seed banks in species with pronounced seasonal dormancy and germination patterns. Seed Sci. Res..

[52] Ozinga W.A., Römermann C., Bekker R.M., Prinzing A., Tamis W.L.M., Schaminée J.H.J., Hennekens S.M., Thompson K., Poschlod P., Kleyer M. (2009). Dispersal failure contributes to plant losses in NW Europe. Ecol. Lett..

[53] Mattana E., Daws M.I., Bacchetta G. (2010). Comparative germination ecology of the endemic *Centranthus amazonum* (Valerianaceae) and its widespread congener *Centranthus ruber*. Plant Spec. Biol..

[54] Peng D., Sun L., Pritchard H.W., Yang J., Sun H., Li Z. (2019). Species distribution modelling and seed germination of four threatened snow lotus (*Saussurea*), and their implication for conservation. Glob. Ecol. Conserv..

[55] Meyer S.E., Kitchen S.G. (1994). Life-history variation in Blue Flax (*Linum perenne*, Linaceae)—Seed-germination phenology. Am. J. Bot..

[56] Rojas-Aréchiga M., Casas A., Vázquez-Yanes C. (2001). Seed germination of wild and cultivated *Stenocereus stellatus* (Cactaceae) from the Tehuacán-Cuicatlán Valley, Central México. J. Arid Environ..

[57] Wilson S.B., Mecca L.K. (2003). Seed production and germination of eight cultivars and the wild type of *Ruellia tweediana*: A potentially invasive ornamental. J. Environ. Hortic..

[58] Maass B.L. (2006). Changes in seed morphology, dormancy and germination from wild to cultivated hyacinth bean germplasm (*Lablab purpureus*: *Papilionoideae*). Genet. Resour. Crop Evol..

[59] Wang M., Li W., Fang C., Xu F., Liu Y., Wang Z., Yang R., Zhang M., Liu S., Lu S. (2018). Parallel selection on a dormancy gene during domestication of crops from multiple families. Nat. Genet..

[60] Ensslin A., Sandner T.M., Matthies D. (2011). Consequences of ex situ cultivation of plants: Genetic diversity, fitness and adaptation of the monocarpic *Cynoglossum officinale* L. in botanic gardens. Biol. Conserv..

[61] Schröder R., Prasse R. (2013). Cultivation and hybridization alter the germination behavior of native plants used in revegetation and restoration. Restor. Ecol..

[62] Ensslin A., Van de Vyver A., Vanderborght T., Godefroid S. (2018). Ex situ cultivation entails high risk of seed dormancy loss on short-lived wild plant species. J. Appl. Ecol..

[63] Therneau T.M. (2020). A Package for Survival Analysis in R. R Package Version 3.1-12. https://CRAN.R-project.org/package=survival.

[64] Onofri A., Gresta F., Tei F. (2010). A new method for the analysis of germination and emergence data of weed species. Weed Res..

[65] Gresta F., Avola G., Onofri A., Anastasi U., Cristaudo A. (2011). When Does Hard Coat Impose Dormancy in Legume Seeds? *Lotus* and *Scorpiurus* Case Study. Crop Sci..

[66] Bradford K.J. (2002). Applications of hydrothermal time to quantifying and modeling seed germination and dormancy. Weed Sci..

[67] Akaike H. (1974). A new look at the statistical model identification. IEEE Trans. Autom. Control.

[68] Rete Stazioni Meteo Linea Meteo 2020. http://www.lineameteo.it/stazioni.php?id=678.

[69] R Core Team (2020). R: A Language and Environment for Statistical Computing, Version 4.0.2.

[70] Pizza R., Espeland E., Etterson J. (2021). Eight generations of native seed cultivation reduces plant fitness relative to the wild progenitor population. Evol. Appl..

