# Supplementary material for: Conditional Seed Dormancy Helps Silene hicesiae Brullo & Signor. Overcome Stressful Mediterranean Summer Conditions
Source: Plants (Basel). 2021 Oct 7;10(10):2130. doi: 10.3390/plants10102130 (PMC8537425; doi:10.3390/plants10102130)
Supplement: Supplementary file 1 [file plants-10-02130-s001.zip › plants-1373564-supplementary.pdf]

Table S1. Final germination percentage (FGP) with standard error (*SE*) for all experimental treatments. Means with no significance letters in common are significantly different ($p < 0.05$). DAH, days afterharvest.

Seed Lot ID	Collection year	DAH	Temperature (°C)	Light Conditions	FGP (%)	SE	Significance letters
PAN17	2017	9	5	D	45	*4.98*	EFGHIJ
				L/D	91	*2.92*	QRSTUVW
			10	D	99	*1.00*	W
				L/D	99	*1.00*	W
			15	D	99	*1.00*	W
				L/D	100	*0.00*	W
			20	D	0	*0.00*	A
				L/D	29	*4.54*	CDEFG
			25	D	0	*0.00*	A
				L/D	0	*0.00*	A
			30	D	0	*0.00*	A
				L/D	0	*0.00*	A
PAN13	2013	260	5	D	50	*5.00*	GHIJKL
				L/D	90	*3.00*	QRSTUVW
			10	D	100	*0.00*	W
				L/D	100	*0.00*	W
			15	D	100	*0.00*	W
				L/D	100	*0.00*	W
			20	D	100	*0.00*	W
				L/D	99	*1.00*	W
			25	D	47	*4.99*	FGHIJK
				L/D	11	*3.13*	ABCD
			30	D	0	*0.00*	A
				L/D	0	*0.00*	A
PAN13	2013	600	5	D	76	*4.27*	KLMNOPQRST
				L/D	97	*1.71*	UVW
			10	D	100	*0.00*	W
				L/D	100	*0.00*	W
			15	D	100	*0.00*	W
				L/D	99	*1.00*	W
			20	D	97	*1.71*	UVW
				L/D	98	*1.40*	VW
			25	D	77	*4.21*	LMNOPQRSTU
				L/D	66	*4.74*	HIJKLMNOPQ
			30	D	0	*0.00*	A
				L/D	0	*0.00*	A
PAN17	2017	600	5	D	80	*4.00*	MNOPQRSTUV
				L/D	98	*1.40*	VW
			10	D	100	*0.00*	W
				L/D	100	*0.00*	W
			15	D	100	*0.00*	W
				L/D	100	*0.00*	W
			20	D	100	*0.00*	W
				L/D	98	*1.40*	VW
			25	D	86	*3.47*	OPQRSTUVW
				L/D	60	*4.93*	HIJKLMNO
			30	D	0	*0.00*	A
				L/D	0	*0.00*	A
PAN17	2017	1000	5	D	37	*4.83*	DEFGH
				L/D	64	*4.80*	HIJKLMNOP
			10	D	99	*1.01*	W
				L/D	100	*0.00*	W

			15	D	100	*0.00*	W
				L/D	100	*0.00*	W
			20	D	100	*0.00*	W
				L/D	99	*1.00*	W
			25	D	88	*3.25*	PQRSTUVW
				L/D	63	*4.83*	HIJKLMNOP
			30	D	0	*0.00*	A
				L/D	0	*0.00*	A
PAN13	2013	2080	5	D	0	*0.00*	A
				L/D	0	*0.00*	A
			10	D	58	*4.94*	GHIJKLMN
				L/D	89	*3.19*	PQRSTUVW
			15	D	92	*2.74*	RSTUVW
				L/D	95	*2.18*	STUVW
			20	D	88	*3.28*	PQRSTUVW
				L/D	94	*2.38*	STUVW
			25	D	77	*4.25*	LMNOPQRSTU
				L/D	60	*4.90*	HIJKLMNO
			30	D	0	*0.00*	A
				L/D	4	*1.96*	AB
FARO19	2019	90	5	D	55	*4.98*	GHIJKLM
				L/D	100	*0.00*	W
			10	D	100	*0.00*	W
				L/D	100	*0.00*	W
			15	D	100	*0.00*	W
				L/D	100	*0.00*	W
			20	D	96	*1.98*	TUVW
				L/D	97	*1.71*	UVW
			25	D	7	*2.55*	ABC
				L/D	9	*2.86*	ABC
			30	D	0	*0.00*	A
				L/D	0	*0.00*	A
FARO19	2019	260	5	D	17	*3.76*	ABCDE
				L/D	99	*1.00*	W
			10	D	99	*1.00*	W
				L/D	99	*1.00*	W
			15	D	100	*0.00*	W
				L/D	100	*0.00*	W
			20	D	98	*1.40*	VW
				L/D	97	*1.72*	UVW
			25	D	64	*4.80*	HIJKLMNOP
				L/D	44	*4.96*	EFGHIJ
			30	D	0	*0.00*	A
				L/D	0	*0.00*	A
FARO17	2017	600	5	D	73	*4.44*	JKLMNOPQRS
				L/D	99	*1.01*	W
			10	D	100	*0.00*	W
				L/D	99	*1.00*	W
			15	D	100	*0.00*	W
				L/D	99	*1.00*	W
			20	D	98	*1.40*	VW
				L/D	100	*0.00*	W
			25	D	99	*1.00*	W
				L/D	93	*2.55*	RSTUVW
			30	D	0	*0.00*	A
				L/D	4	*1.96*	AB
FARO17	2017	1000	5	D	42	*4.94*	EFGHI
				L/D	84	*3.67*	NOPQRSTUVW
			10	D	100	*0.00*	W

				L/D	100	*0.00*	W
			15	D	99	*1.01*	W
				L/D	99	*1.01*	W
			20	D	100	*0.00*	W
				L/D	99	*1.03*	W
			25	D	96	*2.00*	TUVW
				L/D	98	*1.41*	VW
			30	D	8	*2.71*	ABC
				L/D	8	*2.71*	ABC
FARO16	2016	1000	5	D	47	*4.99*	FGHIJK
				L/D	98	*1.40*	VW
			10	D	96	*1.96*	TUVW
				L/D	98	*1.40*	VW
			15	D	96	*1.96*	TUVW
				L/D	98	*1.41*	VW
			20	D	97	*1.71*	UVW
				L/D	99	*1.00*	W
			25	D	88	*3.25*	PQRSTUVW
				L/D	70	*4.58*	IJKLMNOPQR
			30	D	0	*0.00*	A
				L/D	0	*0.00*	A
FARO15	2015	1370	5	D	18	*3.91*	BCDEF
				L/D	41	*4.92*	EFGHI
			10	D	96	*1.96*	TUVW
				L/D	98	*1.40*	VW
			15	D	99	*1.00*	W
				L/D	98	*1.40*	VW
			20	D	98	*1.40*	VW
				L/D	100	*0.00*	W
			25	D	100	*0.00*	W
				L/D	85	*3.57*	NOPQRSTUVW
			30	D	0	*0.00*	A
				L/D	1	*1.00*	AB
PAN17	2017	9	15/10	D	100	*0.00*	W
				L/D	100	*0.00*	W
			20/10	D	100	*0.00*	W
				L/D	100	*0.00*	W
			20/15	D	100	*0.00*	W
				L/D	100	*0.00*	W
			25/20	D	0	*0.00*	A
				L/D	4	*1.96*	AB
PAN13	2013	770	15/10	D	96	*1.96*	TUVW
				L/D	93	*2.58*	RSTUVW
			20/10	D	94	*2.38*	STUVW
				L/D	98	*1.40*	VW
			20/15	D	95	*2.18*	STUVW
				L/D	98	*1.40*	VW
			25/20	D	93	*2.55*	RSTUVW
				L/D	47	*4.99*	FGHIJK

Table S2. Recovery trials. The seeds ungerminated under the first incubation temperature (T1 = 5 °C, 20 °C, 25 °C and 30 °C) were re-incubated, under the same light conditions, at the optimal temperature (T2 = 15 °C). DAH, days after harvest; FGP, final germination percentage at T1, T2 and their sum (T1 and T2); *SE*, standard error.

Seed Lot ID	T1 (°C)	Light Conditions	DAH	FGP T1 (%)	*SE*	Re-incubation Temperature T2 (°C)	FGP T2 (%)	*SE*	Sum of FGPs (T1 and T2) (%)	*SE*
PAN17	20	L/D	9	29	*4.54*	15	67	*4.70*	96	*1.96*
PAN17	25	L/D	9	0	*0.00*	15	96	*1.96*	96	*1.96*
PAN17	30	L/D	9	0	*0.00*	15	95	*2.18*	95	*2.18*
PAN17	30	L/D	600	0	*0.00*	15	100	*0.00*	100	*0.00*
PAN17	30	D	600	0	*0.00*	15	94	*2.37*	94	*2.37*
PAN 17	30	L/D	1000	0	*0.00*	15	100	*0.00*	100	*0.00*
PAN17	30	D	1000	0	*0.00*	15	100	*0.00*	100	*0.00*
PAN13	25	L/D	260	11	*3.13*	15	87	*3.36*	98	*1.40*
PAN13	25	D	260	47	*4.99*	15	50	*5.00*	97	*1.71*
PAN13	30	L/D	260	0	*0.00*	15	99	*0.99*	99	*0.99*
PAN13	30	D	260	0	*0.00*	15	94	*2.37*	94	*2.37*
PAN13	5	L/D	2080	0	*0.00*	15	90	*3.00*	90	*3.00*
PAN13	5	D	2080	0	*0.00*	15	82	*3.84*	82	*3.84*
PAN13	30	L/D	2080	4	*1.96*	15	82	*3.84*	86	*3.47*
PAN13	30	D	2080	0	*0.00*	15	81	*3.92*	81	*3.92*
FARO19	25	L/D	90	11	*3.13*	15	89	*3.13*	100	*0.00*
FARO19	25	D	90	7	*2.55*	15	91	*2.86*	98	*1.40*
FARO19	30	L/D	90	0	*0.00*	15	100	*0.00*	100	*0.00*
FARO19	30	D	90	0	*0.00*	15	96	*1.96*	96	*1.96*
FARO19	25	L/D	260	44	*4.96*	15	54	*4.98*	98	*1.40*
FARO19	25	D	260	64	*4.80*	15	35	*4.77*	99	*0.99*
FARO19	30	L/D	260	0	*0.00*	15	99	*0.99*	99	*0.99*
FARO19	30	D	260	0	*0.00*	15	98	*1.40*	98	*1.40*
FARO17	30	L/D	600	4	*1.96*	15	89	*3.13*	93	*2.55*
FARO17	30	D	600	0	*0.00*	15	90	*3.00*	90	*3.00*
FARO17	30	L/D	1000	8	*2.71*	15	91	*2.86*	99	*0.99*
FARO17	30	D	1000	8	*2.71*	15	92	*2.71*	100	*0.00*
FARO16	30	L/D	1000	0	*0.00*	15	97	*1.71*	97	*1.71*
FARO16	30	D	1000	0	*0.00*	15	75	*4.33*	75	*4.33*
FARO15	30	L/D	1370	1	*0.99*	15	96	*1.96*	97	*1.71*
FARO15	30	D	1370	0	*0.00*	15	97	*1.71*	97	*1.71*
